# Deltas’ and spindles’ cross-area synchronization and ripple subtypes

**DOI:** 10.1093/sleep/zsag168

**Published:** 2026-06-19

**Authors:** Adrian Aleman-Zapata, Yixiao Zhang, Pelin Özsezer, Kopal Agarwal, Abdelrahman Rayan, Irene Navarro-Lobato, Lisa Genzel

**Affiliations:** Donders Institute for Brain, Cognition and Behaviour, Radboud University, Postbus 9010, 6500GL Nijmegen, The Netherlands; Donders Institute for Brain, Cognition and Behaviour, Radboud University, Postbus 9010, 6500GL Nijmegen, The Netherlands; Donders Institute for Brain, Cognition and Behaviour, Radboud University, Postbus 9010, 6500GL Nijmegen, The Netherlands; Donders Institute for Brain, Cognition and Behaviour, Radboud University, Postbus 9010, 6500GL Nijmegen, The Netherlands; Donders Institute for Brain, Cognition and Behaviour, Radboud University, Postbus 9010, 6500GL Nijmegen, The Netherlands; Donders Institute for Brain, Cognition and Behaviour, Radboud University, Postbus 9010, 6500GL Nijmegen, The Netherlands; Donders Institute for Brain, Cognition and Behaviour, Radboud University, Postbus 9010, 6500GL Nijmegen, The Netherlands

**Keywords:** hippocampal ripples, memory consolidation, sleep, prefrontal cortex, delta waves, spindles, neural synchronization

## Abstract

Hippocampal ripples, critical for sleep-related memory consolidation, are heterogeneous events with various sources and functions. However, their specific roles in supporting different forms of memory remain poorly understood. Here, we applied principal component analysis to identify ripple sub-types and relate them to hippocampal–cortical interactions, as well as their role in consolidating simple and complex semantic-like memories in rats. Three main ripple types were identified: *medium-sized, large-sized*, and *small-sized* ripples. *Small-sized* ripples were associated with increased prefrontal cortex to hippocampus connectivity, followed hippocampal delta waves, and were related to simple learning. In contrast, *large-sized* ripples exhibited increased hippocampus to prefrontal cortex connectivity, occurred during hippocampal spindles together as a doublet with a *small-sized* ripple, and were related to complex memory consolidation. Finally, learning induced heightened coupling between hippocampal delta and spindle oscillations and their cortical counterparts, consequently leading to an increased synchronization of ripples with cortical oscillations. Together, these results underscore that distinct ripple subtypes and their specific patterns of hippocampal–cortical coordination during sleep underpin different memory consolidation processes.

## Graphical Abstract

**Graphical abstract:**
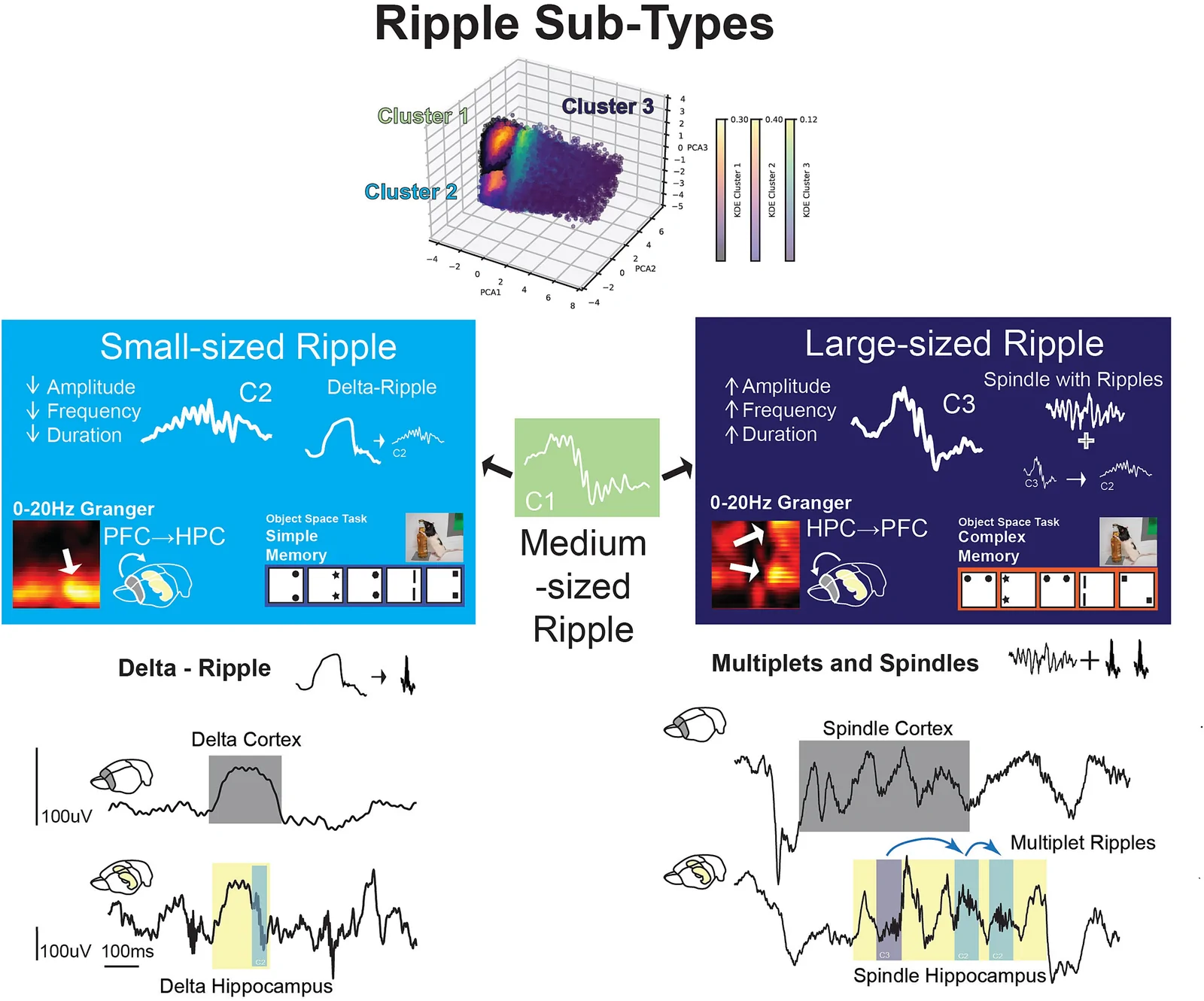

**Statement of Significance**

Our data-driven analysis identifies distinct ripple subtypes and links them to specific patterns of hippocampal–cortical interaction, building on and extending prior reports of ripple heterogeneity and hippocampal–cortical coordination to show how different ripple groups may support memory consolidation.

## Introduction

Hippocampal ripple oscillations are critical for memory consolidation [1, 2] and underlie the beneficial effect of sleep on memory [3]. The spiking content in the CA1 hippocampal layer during ripples is influenced by various brain regions [4–6] with inputs from the hippocampal CA3 and CA2, as well as the medial entorhinal cortex, shown to influence ripple activity [7–10]. However, it remains unclear how distinct ripple subtypes map onto the various cognitive processes associated with ripple activity.

Ripples exhibit significant diversity in their characteristics, suggesting a heterogeneous event arising from multiple contributions. Ripple subtypes can be determined by employing different supervised and unsupervised methods. The simplest supervised method classifies ripples by thresholding a ripple feature (e.g. short- vs long-duration ripples [11–14]) or categorizing ripples based on percentiles of a given feature [15, 16]. A second supervised alternative determines ripple subtypes based on the cooccurrence of the hippocampal ripple with other oscillations, such as cortical ripples [3, 17], spindles and delta waves [14, 18], and sharp-waves [13]. Recent studies have demonstrated that unsupervised learning techniques and threshold-based approaches can be employed to disentangle this inherent diversity [19–22]. Using the local field potential trace of the ripple, these methods can differentiate ripple types based on their amplitude, frequency, duration and other features and it has been shown that these ripple types relate to differences in input sources [13, 19].

Previous efforts have successfully identified different types of ripples but have not established their functional relevance [19, 21]. To elucidate this relationship, ripple clustering should be performed in experiments involving relevant behavioural manipulations and their corresponding readouts. In this study, we detected and clustered ripples occurring during sleep following different learning conditions, compared to home cage controls, using the Object Space Task (OST) [23]. Further, we included an experimental group in which we manipulated cortical plasticity to elucidate top–down cortical control of ripple types [4].

In this study, we cluster hippocampal ripples into three major subtypes and examine how each relates to cortical firing patterns, oscillatory coupling (delta waves and spindles), and memory consolidation. Beyond the prevalent medium-sized pattern, we identify *small-sized ripples*—low-amplitude, short-duration events that follow hippocampal delta waves and are accompanied by increased prefrontal–hippocampal connectivity, occurring more after simple learning (reinforced information across trials). In contrast, *large-sized ripples* are longer, high-amplitude events nested within hippocampal spindles, show strong hippocampal→prefrontal connectivity, and occur more after complex memory consolidation (changing information from trial to trial). Notably, both non-medium-sized subtypes tend to occur in bursts after complex learning, suggesting that coordinated sequences of ripples support hippocampal–cortical dialogue during sleep.

## Materials and methods

### Study design

A total of eight rats were used in this experiment, which we have previously analyzed with a different emphasis and published [4]. One vehicle animal was excluded from this ripple analysis, since we did not detect ripple in the simple learning condition and only included animals that had a fully balanced data set. Animals were first extensively handled for multiple days (at least three) until they did not flinch when the experimenter touched them (see handling videos on www.genzellab.com). Next, all animals underwent viral-injection surgery (see below); half the animals received RGS14_414_-lentivirus, while the other had a vehicle (empty) lentivirus. The eight electrophysiology animals (four vehicle, four RGS14) received a second surgery 3 weeks after the first one, for hyperdrive implantation. During 2–3-week surgery recovery, tetrodes were slowly lowered to target area before the animals also had habituation and training in the OST.

### Animals

Three-month-old male Lister Hooded rats weighing between 300 and 350 g at the experiment start (Charles Rivers, Germany) were used in this study. Rats were pair-housed in conventional eurostandard type IV cages (Techniplastic, UK) in a temperature-controlled (20 + 2°C) room following a 12-h light/dark cycle with water and food provided *ad libitum*. After lentiviral surgical intervention, animals were single-housed for 2 days and paired with their cage mates after recovery in rat individually ventilated cages (IVCs; Techniplastic, UK). Animals were maintained in their IVCs in a barrier room for 14 days before downscaling them to conventional housing conditions. After hyperdrive implantation, rats were single-housed in until the end of the experiment. A total of eight rats were in the electrophysiological recordings (*n* = 4 per treatment, RGS14_,_ and vehicle, split one animal per treatment across four cohorts). The behavioral experiments and electrophysiological recordings were performed during the light period (between 9:00 and 18:00).

All animal procedures were approved by the Central Commissie Dierproeven and conducted according to the Experiments on Animals Act (protocol codes, 2016-014-020 and 2016-014-022).

### RGS14414-lentivirus

The RGS14_414_- and vehicle-lentivirus solutions (1.72 × 10^7^ CFU/mL and 2.75 × 10^6^ CFU/mL, respectively) were prepared and provided by Dr. Zafaruddin Khan at the University of Malaga (Malaga, Spain) [24, 25]. Briefly, the RGS14_414_ gene (GenBank, AY987041) was cloned into the commercial vector p-LVX DsRed Monomer-C1 (Clontech, France) using DNA recombinant technology. Then, both non-replicant RGS14_414_- and vehicle-lentivirus (empty vector) were prepared and titered using the Lenti-XTM (Clontech, France) according to the manufacturer’s instructions.

The animal’s procedures related to the non-replicant lentiviral solution were approved and carried out in compliance with institutional regulation.

### Tetrode hyperdrive

A customized lightweight tetrode micro-drive was manufactured to implant 10 and 6 movable tetrodes in the prelimbic cortex and hippocampus (HPC), respectively [26–28]. Two separate bundles of #33 polyimide tubes (Professional Plastics) were prepared: one of 2 columns × 5 rows for prelimbic cortex and 3 × 3 for HPC. The bundles were fixed first to the customized 3D-printed cannula and then into the customized 3D-printed body drive. The 3D-printed cannula was designed according to the Rat Brain Atlas in Stereotaxic Coordinates [29] for the correct placement of the bundles in the areas of interest. Inner tubes (#38 polyimide tubes; Professional Plastics) were placed inside the outer tubes and glued to the shuttle, which moves through the body spokes thanks to an inox steel screw and a spring CBM011C 08E (Lee Spring, Germany). A total of 16 tetrodes were built, twisting four 10 cm polyimide-insulated 12 μm Nickel-Chrome wires (80 turns forward and 40 turns reverse) (Kanthal Precision, Florida) and fused by heat. Tetrodes were loaded in the inner tubes, and their free ends were connected to a customized 64 channels, 24 mm round electrode interface board (EIB) using gold pins (Neuralynx). Previously, two NPD dual row 32 contact connectors (Omnetics) had been attached to the EIB. The tetrode tips were cut using fine sharp scissors (maximum length 3.5 and 3 mm for prelimbic cortex and HPC, respectively) and fixed to the inner tubes in the upper part. Tetrode tips were clean in distilled water and gold-plated (gold solution, Neuroalynx) using NanoZ software to lower their impedance to 100–200 kΩ and improve the signal-to-noise ratio. The tetrode tips were hidden at the same level as the bundle. The whole drive was covered with aluminum foil connected to the ground to reduce the electrostatic interference during the recordings. The bottom of the micro-drive was deepened in 70% ethanol for 12 hours before brain implantation.

### Stereotaxic surgeries

#### Lentivirus injection

Lentiviral solutions were infused in the prelimbic cortex using stereotaxic surgery under biosafety level 2 conditions. The coordinates of the prelimbic cortex injection site were + 3.2 mm AP, +/−0.8 mm ML from Bregma, and −2.5 mm DV from dura mater, according to The Rat Brain Atlas from Paxinos and Watson [29]. The procedure was carried out under isoflurane inhaled anesthesia. Unconsciousness was induced at 5 % isoflurane +1 L/min O_2_ and maintained at 1.5%–2% isoflurane +1 L/min O_2_. A 0.8 mm–diameter craniotomy was drilled above the target area in each hemisphere. The DV dura mater coordinate was measured before performing the durotomy.

A 30 G dental carpule connected to a 10 μL Hamilton and an infusion pump (micro-pump, WPI) was slowly inserted into the brain target area (0.2 mm/min). A total volume of 2 μL of the lentiviral solution was infused at 200 nL/min. After 5 minutes of diffusion, the needle was removed, and the incision was sutured.

Temperature, oxygen saturation, and blood pressure were monitored during the whole surgical procedure. Some eye cream (Opthosam) was applied to protect the corneas during the intervention. At the start and end of the surgery, 2 mL of 0.9% NaCl physiological serum was administered subcutaneously. As analgesia, animals were administered 0.07 mg/mL carprofen in their water bottles 2 days before and 3 days after surgery. Immediately before surgery, 5 mg/kg carprofen was sc-injected. In addition, a mix of 4 mg/kg lidocaine and 1 mg/kg bupivacaine in a 0.9% NaCl physiological serum was administered sc locally in the head.

After the viral injection, animals were housed individually in rat IVC cages for 14 days. Their weights and status were monitored daily for the correct recovery of animals. Then, rats were pair-housed with their previous cagemate and moved to conventional housing.

### Tetrode hyper-drive implantation

Twenty-one days after viral infusion, a second stereotaxic surgery took place for tetrode micro-drive implantation in eight animals. The procedure was similar as described above. In this intervention additionally, a prophylactic 10 mg/kg sc injection of Baytril antibiotic was administered at the beginning of the surgery. Two craniotomies (2 × 1 mm and 1 × 1 mm for prelimbic cortex and HPC, respectively) were drilled above the target areas on the right hemisphere. The coordinates for the upper-left corner of each craniotomy were: AP +4.5 mm and ML −0.5 (prelimbic cortex) and AP −3.8 mm and ML −2 mm (HPC) from Bregma [4]. A ground screw (M1 × 3) was placed on the left hemisphere in the cerebellum (AP −11 mm, ML +2 mm from Bregma). In addition, six M1 × 3 mm supporting screws were driven and bound to the skull using Super-bond C&B dental cement (Sun Medical, Japan). Carefully, the durotomies were performed, and the brain’s surface was exposed. Subsequently, the micro-drive was positioned on the brain’s surface and attached to the skull and the screws by simplex rapid dental cement (Kemdent, UK). Then, tetrodes were slowly screw-driven into the prelimbic area in prelimbic cortex (3 mm DV from brain surface) and the cortical layers above the HPC (1.5 mm DV from brain surface). The dorsal hippocampal CA1 pyramidal layer was reached progressively in the subsequent days.

### Object Space Task

The OST is a newly developed behavioral paradigm to study simple and semantic-like memories in rodents [23]. The task is based on the tendency of rodents to explore novel object location in an open field across multiple trials. In these experiments, the OST took place as described previously [23] at least 21 days after the viral infusion when the effect of the RGS14 protein is observed [24,25]. Briefly, animals were handled for five consecutive days before and after surgery recovery. Then, rats were accommodated in the experimental room and habituated to the open field across five sessions (one per day). In the first session, animals explored the open field with their cagemate for 30 minutes. In the rest of the session, each individual freely explored the open field for 10 min. Two Duplo objects were included in the open field center in the last two sessions to facilitate a better exploration time in the subsequent task.

The OST consists of two phases: a training phase of five training trials in which animals are exposed to two identical objects (different across trials) for 5 minutes (45–55 min intertrial time); and an interference/test phase consisting of a single 10-min trial performed 24 and 72 h after training. In the stable condition, both object locations were fixed during the training trials, and one object location was moved during the interference and tests sessions. In the overlapping condition, one object location was fixed, and the other one moved across training trials. In the interference and test sessions, the same object-location pattern from the last training trial was repeated.

The open field was a wooden square 75 × 75 × 60 cm. For the task, but not for the habituation, we placed 2D proximal cues on the open field walls and 3D distal cues above the open field. The cues were changed in each experimental session. In addition, the open field base colors changed across task sessions (white, blue, green, brown). The objects used vary in material (plastic, glass, wood, and metal), size, and colors. For electrophysiological recordings, not plastic objects were used to prevent static interference. All object bases were attached to 10 cm × 10 cm metal plates. Circular magnets were installed in the corners underneath the open field floor to prevent object movements during exploration. The open field and object surfaces were cleaned with 40% ethanol between trials to avoid odor biases.

Each trial was recorded using a camera above the open field. The object exploration time was manually scored *online* using the homemade software “*Scorer32.*” The experimenter was blinded for treatment and experimental conditions at the moment of scoring. Object locations and experimental conditions were counterbalanced across treatments, individuals, and sessions.

For electrophysiological recordings, we run four cohorts of two animals each. Each cohort included one vehicle- and one RGS14-treated animal, which performed the identical condition sequences with the same object-location patterns. Object locations and experimental conditions were counterbalanced across cohorts. Electrophysiological recordings took place during trials and rest periods (45 min before and 3 h after both training and test; and 45-min intertrial time during the training). Therefore, two brown wooden sleep boxes (40 × 75 × 60 cm height) with bedding material were placed next to the open field. Animals had been accustomed to the sleep box for at least 3 h in each open field habituation session.

Rats involved in the electrophysiological recordings also performed two experimental control conditions: home cage and random. The random condition was carried out as described previously [23], so there was a lack of repetitive object location patterns across different trials. In the home cage, the animal was recorded for 7 h and 10 min in the sleep box (a whole training session recording), and the experimenter kept the rat awake for the equivalent trial times.

### In vivo electrophysiology recordings

In vivo freely moving extracellular recordings were executed during the OST and the resting periods. One session per experimental condition (home cage, stable, overlapping, and random) was carried out per animal. The local field potential (LFP) and single-unit activity detected by the 64 channels were amplified, filtered, and digitized through two 32-channel chip amplifier headstages (InstanTechnology) connected through the Intan cables and a commutator into the Open Ephys acquisition box. The signal was visualized using the open-source Open Ephys GUI (sample rate 30 kHz). In addition, the headstage contained an accelerometer to record the movement of the animals.

### Sleep scoring

Different states (wakefulness, Rapid Eye Movement [REM], NonREM, and Intermediate) were off-line manually scored using, “TheStateEditor” developed by Dr. Andres Grosmark at Dr. Gyorgy Buzsaki’s lab. One channel per brain area (Prelimbic cortex and hippocampus) was selected per animal. Using a 10-s sliding window, an experienced researcher created the hypnogram and 1-s epoch vector indicating brain states. The absences of movement in the accelerometer discriminate between wakefulness and sleep. During sleep periods, a dominant theta frequency in the dorsal hippocampus in the absence of spindles and delta waves indicated REM sleep. NonREM sleep was classified when slow oscillations were detected in the prelimbic cortex. The intermediate phase was defined as short transitional periods between NonREM and REM that show an increase in frequency in the prelimbic cortex and frequency similar to theta in the dorsal hippocampus. Microarousals were defined later as periods of wakefulness <15 s within a sleep period.

### Signal preprocessing

For the following analyses, first a single channel was selected per brain area. For prelimbic cortex, the channel with the largest slow oscillations was chosen. For hippocampus, the channel closest to the pyramidal layer, which displayed noticeable ripples was selected. Both channels were originally acquired at a sampling rate of 30 kHz, and to avoid working which such a high rate, the channels were filtered with a third-order Butterworth lowpass filter at 500 Hz to avoid signal aliasing and then downsampled to 1 kHz.

### Detection of spindles and delta waves

The downsampled prelimbic cortex channel (1 kHz) was loaded into the Matlab workspace, and using a third-order Butterworth filter, the signal was filtered to 9–20 Hz for detecting spindles and to 1–6 Hz for detecting delta waves. The NonREM bouts were then extracted from the filtered signal and concatenated. The functions FindSpindles and FindDeltaWaves from the Freely Moving Animal (FMA) toolbox http://fmatoolbox.sourceforge.net were modified and used to detect the start, peak, and end of spindles and delta waves, respectively. The optimal threshold was found for each animal by visually inspecting the detections and modifying the default parameters of the functions when needed. The results were saved as timestamps with respect to the concatenated NonREM signal in seconds. They were then used to find the timestamps with respect to the recorded signal. This process was repeated for pre- and post-trial sleep periods in study days pertaining to all animals in both treatment groups. The same method was applied for the detection of hippocampal delta waves.

### Ripple detection

**Figure 1:**
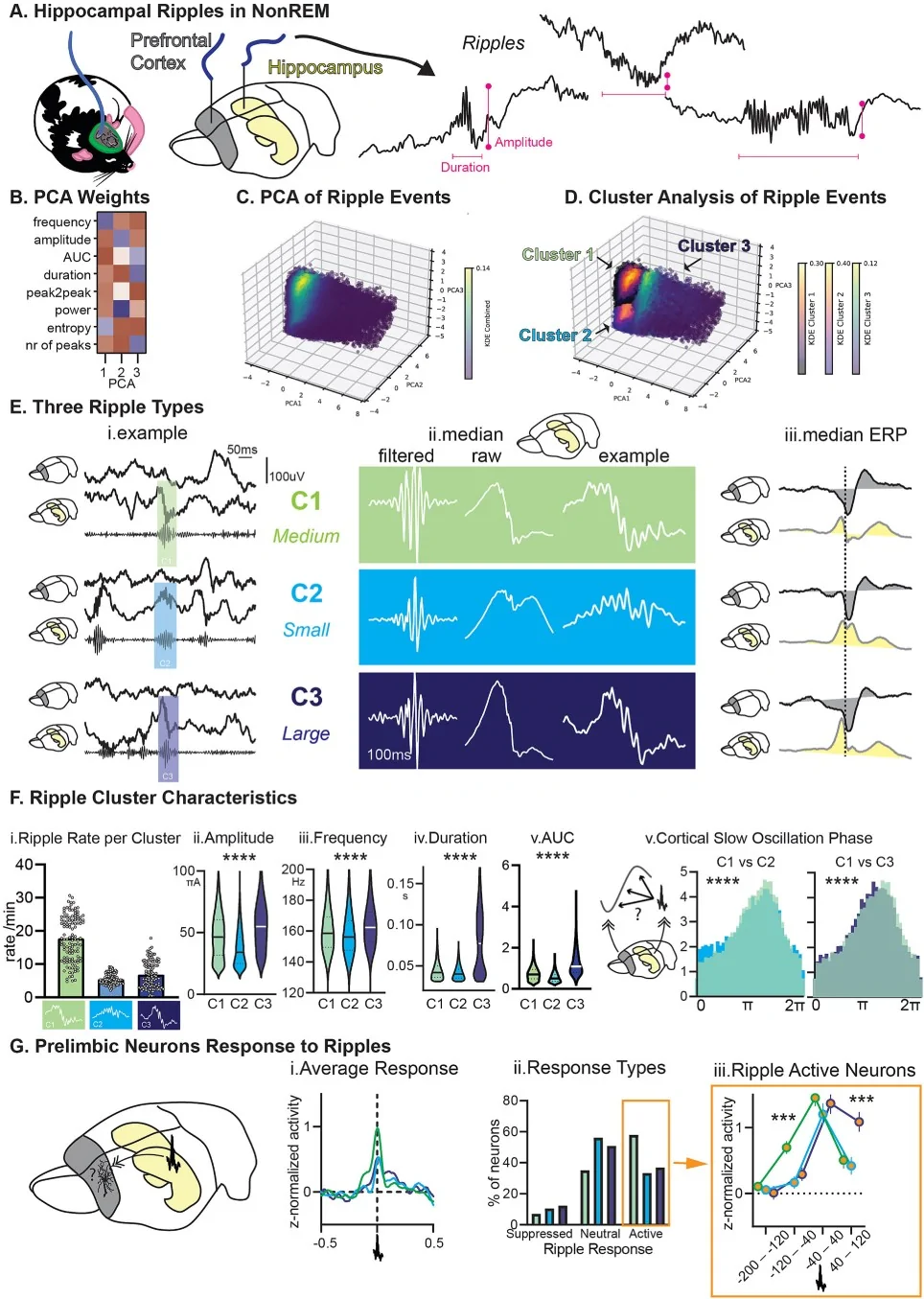
Ripple subtypes: (A) We recorded sleep and detected ripples in the hippocampus. Ripples come in many shapes and forms. (B) Features and their weights in each PC (principal component) (C) PCA on ripple characteristics showed three main clusters (D) Determined with a GMM clustering algorithm and evaluated with the Calinski–Harabasz index and its consensus with Akaike and Bayesian information criteria. See Video S1. The colorbar shows the kernel density estimation (KDE) of ripple occurrence based on PCA1 and PCA2, which capture most of the data variability, displayed for all ripples together and for each cluster individually. The discarded cluster with few events is not shown. (E) Representative and median traces for each cluster. (Ei) Examples of individual ripple events (raw and filtered) and corresponding cortical traces. (Eii) Median event-related potentials (ERPs) of raw and filtered traces aggregated across all ripple events and all animals, along with a single example trace for reference. C1 and C3 are noticeable with larger deflection potentially volume conducted sharp-wave. Traces have been normalized for visualization, showing similar waveforms for C1 and C3. However, non-normalized amplitudes were used in the PCA, and results indicate that C3 ripples had higher amplitudes compared to C1. (Eiii) Raw traces from (Eii) displayed for hippocampus and prefrontal cortex to highlight interregional dynamics. Median data correspond to each ripple subtype, aggregated across all animals. (F) Characteristics for ripples from each cluster (individual events aggregated across all animals: Cluster 1: 28 857 events; Cluster 2: 8525 events; Cluster 3: 11 039 events). Most ripples were part of C1 (i, including PT periods rmANOVA type *F*_2,186_ = 404.1 *p* < .0001). Subtypes differed in amplitude (ii, including events Kruskal–Wallis = 6427 *p* < .0001), frequency (iii, including events Kruskal–Wallis = 1165 *p* < .0001), duration (iv, including events Kruskal–Wallis = 11 464 *p* < .0001), area under the curve (v AUC, each KS-D *p* < .0001), and slow oscillations phase coupling (vi, each KS-D *p* < .0001). (G) Response of prelimbic neurons to ripple subtypes (ii): Percentage of ripple-active, neutral or suppressed neurons computed across the full sample. (iii): Response at baseline (200–120 ms before ripple), pre-ripple (120– 40 ms before ripple), during ripple (40 ms before–40 ms after ripple peak), and post-ripple (40–120 ms after ripple). Cluster 1 had a higher ripple response in prelimbic neural activity (rmANOVA cluster^*^time *F*_196,10 976_ = 3.1 *p* < .001), marginally more ripple-active neurons (chi-square 8.425 *p* = .077) and higher average activity of ripple-active neurons before the ripple (rmANOVA cluster^*^time *F*_6,210_ = 4.7 *p* < .001, cluster 1 vs other pre *F*_1,71_ = 15.8 *p* < .001). Cluster 3 had higher average neuron activity after the ripple (cluster 3 vs other post *F*_1,69_ = 14.2 *p* < .001). ^*^p < .05, ^**^p < .01, ^***^p < .001, ^****^p < .0001; all error bars are SEM. ERP, event-related potential; AUC, area under the curve.

The downsampled channels (1 kHz) of the hippocampal pyramidal layer were loaded into the Matlab workspace, and the NonREM bouts were extracted. Using a third-order Butterworth bandpass filter, the epochs of HPC signal were filtered to a frequency range of 100–300 Hz. A custom MATLAB function was used for detecting the start, peak, and end of the ripples by thresholding voltage peaks that lasted a minimum duration of 30 ms above the threshold. The start and end of the ripple were determined as half the value of the selected threshold. Values equivalent to five times the standard deviation of concatenated NonREM bouts were computed individually for presleep and all post-trials in a study day. The average of these values was calculated to find a single detection threshold per study day. An offset of 5 μV was added to the threshold to reduce false positives. This was repeated for all study days pertaining to all animals in both treatment groups. To identify consecutive ripple events (multiplets), ripple peaks occurring within 200 ms of each other were grouped and classified as doublets, triplets, and so on, with their timestamps stored for further analysis. Since cluster 3 had a very large spread of ripple durations (Figure 1F  iv), which showed a bimodal distribution, we split this cluster into short (≤70 ms) and long events (>70 ms) for the multiplet-spindle analysis (according to the bimodal distribution). The shorter duration ripples are short enough to occur during one spindle duty cycle.

### Dimensionality reduction of ripple features and ripple clustering

Once ripples were detected, we extracted their waveforms (sampling rate = 1 kHz) using the start and end timestamps given by the detector. Instead of using a fixed temporal window, we computed ripple features using the intrinsic duration of each ripple waveform. Ripple features were computed as follows.

#### Average frequency

The Matlab function meanfreq() was used to calculate the mean frequency of ripples. It takes in two inputs, the signal and the sample rate, and estimates the mean normalized frequency of the signal’s power spectrum. First, the power spectral density of the signal is computed using the periodogram() function and a default Kaiser window. Then, the mean frequency is estimated as a weighted average, using the power spectral density values as weights of their corresponding frequencies.

#### Amplitude

To calculate the amplitude of the signal the envelope was computed by applying Hilbert transform. Then, the absolute value of this transform was taken to obtain real values. Finally, the maximum value of the envelope was calculated.

#### Area under the curve

The Matlab function trapz() was used to calculate the area under the curve of ripple waveforms absolute values. This function numerically integrated the absolute value of each ripple waveform over its duration in seconds.

#### Duration

The duration of each ripple was calculated in seconds using the start and end timestamps obtained from the ripple detector. As mentioned in the ripple detection section, the start and end of the ripple were determined as half the value of the amplitude detection threshold.

#### Peak-to-peak distance

The Matlab function peak2peak() was used to calculate the peak-to-peak distance. This function calculates the difference between the maximum and minimum amplitude values of the ripple waveform.

#### Power

The power of a ripple was computed as the squared Euclidean norm (i.e. vector magnitude) of a ripple waveform divided by its length in samples.

#### Entropy

The Matlab function entropy() was used to calculate the entropy of each ripple. Entropy is defined as -sum(p.^*^log2(p)), where p represents the normalized histogram counts.

#### Number of peaks

The Matlab function findpeaks() was used to find the local maxima (i.e. peaks), defined as data samples larger than its two neighboring samples without a minimum prominence. The total number of peaks was computed per ripple.

Each feature array was scaled using a z-score normalization. We performed principal component analysis (PCA) on the ripple features of aggregated ripples. For RGS14 ripples, we used the PCA coefficient obtained from vehicle ripples to project the data into the same space and allow comparisons between treatments. We used the first three principal components that explained most of the variance. The resulting tridimensional point cloud was clustered using a Gaussian Mixture Model (GMM). Only data from the vehicle animals were used for the initial cluster analysis. To find the optimal number of clusters, we employed a consensus approach. Akaike information criterion (AIC) and Bayesian information criterion (BIC) were calculated on a GMM ranging from 1 cluster to 10 clusters. The resulting information criterion curves began to plateau around four to five clusters. To verify further, the optimal number of clusters was calculated using *k*-means clustering and the Calinski–Harabasz criterion [30]. The result proved to be four, which mitigated the risk of overfitting. Therefore, the number of clusters was set to 4. Finally, the built-in MATLAB function fitgmdist() was used to fit a GMM to the data. The parameter “CovarianceType” was set to “diagonal.”

The procedure was repeated with fixed PCA coefficients obtained from vehicle ripples applied to the data from RGS14 group. Clustering was performed separately for these ripples.

### Kernel Density Estimation in 3D PCA space

We computed Kernel Density Estimation (KDE) to visualize the density of clusters in a 3D PCA space.

For each identified cluster, we extracted the 3D coordinates of its points in PCA space. We then applied a Gaussian KDE using the first two components (PCA1 and PCA2), as these account for the largest variance. The estimated densities were mapped to colors in a 3D scatter plot, with PCA3 providing the third spatial dimension. Colormaps were chosen to maximize contrast and interpretability within the limitations of the Python matplotlib library. All KDE computations were performed using the gaussian_kde function from scipy.stats, and visualizations were implemented with matplotlib in Python.

### Oscillation characteristics

The traces of each event detected (ripples, spindles, delta waves) were extracted using the start and end timestamps obtained from the detectors. The traces of the events were filtered in their corresponding detection frequency band. Characteristics such as the amplitude and mean frequency were calculated for these filtered events using built-in and custom MATLAB functions. Namely, the amplitude of the events was calculated by computing the envelope of the filtered trace using a Hilbert transform. The absolute value of the result was taken, and its maximum was found. The mean frequency of the filtered traces was computed using the meanfreq function of MATLAB.

### Detection of oscillation sequences

The sequences between ripples, spindles, and delta waves were counted in various combinations to study cortico-hippocampal coupling during NonREM sleep as done by Maingret et al. [18]. The time difference between the peaks of these events was compared to a fixed duration to establish if there was a sequential relationship in the following combinations of oscillations: Delta-Spindle (D-S), Delta-Ripple (D-R), Ripple-Delta (R-D), Ripple-Delta-Spindle (R-D-S), and Delta-Ripple-Spindle (D-R-S). For D-S, a sequence was considered when the duration between events was between 100 and 1300 ms; for D-R, it was 50–400 ms, and for R-D, it was 50–250 ms. To find R-D-S sequences, the results of R-D and D-S were compared to find delta waves preceded by a ripple and followed by a spindle. To find D-R-S sequences, the results of D-R and R-S (events between 2 and 1000 ms) were compared to find ripples preceded by a delta wave and followed by a spindle. To find the Delta-Spindle with Ripple sequences, the results of D-S and spindles co-occurring with ripples (see next subsection) were matched to find spindles preceded by a delta and co-occurring with a ripple. The results were saved as counts of each sequence for each post-trial.

### Co-occurrence between ripples and spindles

The co-occurrence between ripples and spindles was computed by comparing the start and end timestamps of both events. To consider co-occurrence between a ripple and a spindle, either one of the following conditions had to be fulfilled. (1) A ripple had to start and end within the duration of the spindle. (2) One of the events had to start or end within the duration of the other. Given that more than one ripple can co-occur with the same spindle, we counted separately spindles co-occurring with spindles and spindles co-occurring with ripples.

### Permutation analysis ripples, spindles, delta

The statistical significance of cluster-specific conditional ripple occurrence was determined by a permutation test. Ripple cluster labels were randomly shuffled, and a conditional occurrence ratio was computed for each permuted dataset. This procedure was repeated 5000 times to generate a permutation-based null distribution, against which the observed cluster-specific ratio was evaluated. To control for imbalance in cluster size, cluster 1 and cluster 3 were resampled to match the size of cluster 2. We further applied bootstrap procedure to generate the 95% confidence interval for the observed ratios, providing an estimate of their uncertainty.

### Slow oscillation phase

The downsampled prelimbic cortex signal was filtered in the 0.5–4 Hz range using a third-order Butterworth bandpass filter, and its Hilbert transform was computed to find the phase angle of slow oscillations in a range from 0° to 360°. The peaks of ripples and spindles were then used to find the corresponding slow oscillation phase. This same signal was later used to find the phase during spikes timestamps of cortical neurons.

### Time–frequency spectrograms

A 2-second-long window centered on each ripple peak was extracted from the hippocampus and the prelimbic cortex channels, respectively. All vehicle ripples across animals and conditions were combined for each ripple cluster, and their amplitude was computed by finding the maximum of their envelope computed with a Hilbert transform. The median ripple amplitude was calculated and the corresponding 2-second-long windows of the 2000 ripples which amplitude was the closest to the median amplitude were included in the following analysis. A notch filter at 50 Hz was applied to the ripple-centered windows using the ft_preprocessing function from the Matlab-based Fieldtrip toolbox [31]. The short-time Fourier transformation was calculated to detect the changes of spectral power in hippocampus and prelimbic cortex with respect to a time window of ±1 s around each ripple. This was computed using the ft_freqanalysis function from Fieldtrip with a 100 ms Hanning window and time steps of 10 ms, for a frequency range from 100 to 300 Hz with a 2 Hz step and from 0.5 to 20 Hz with a step of 0.5 Hz. The resulting spectrograms were averaged and displayed. To statistically compare spectrograms between ripple clusters, a non-parametric permutation test to correct for multiple comparisons with two-tailed pixel-based statistics was computed using 500 permutations and a *P*-value of 0.05 [32].

### Time–frequency Granger causality

To determine the predictive power between brain regions during ripples, the time–frequency Spectral Granger Causality was computed for each directionality [33]. A window with length of 2.2 s centered around each ripple peak was extracted for the simultaneous hippocampal and prelimbic cortex signals. The length of this window was chosen to at least capture one cycle of 0.5 Hz activity. A 2-second-long time–frequency non-parametric Spectral Granger causality was computed by implementing a short-time Fourier transform with a 500-ms Hanning window with 10-ms steps using the Fieldtrip functions ft_freqanalysis and ft_connectivityanalysis, respectively. To determine statistical differences between Granger spectrograms, we created randomized trials by taking 400 random ripples per ripple cluster and computing their time–frequency Granger causality as described above. The result was stored, and the procedure was repeated 30 times to give a total of 30 randomized trials per ripple cluster. We then used the trials of the ripple clusters to determine significant statistical difference in each pixel of the time–frequency matrix by applying a non-parametric permutation test to correct for multiple comparisons with a two-tailed pixel-based correction, using 500 permutations and a *P*-value of 0.05.

### Spike sorting

An important step of our analysis was identifying the cortical neurons and their spiking activity. The sleep and trial recordings of each tetrode of the prelimbic cortex were extracted and concatenated chronologically to allow tracking the individual neuronal activity across the whole study day. The tetrode recordings were preprocessed by applying a bandpass filter between 300 and 3000 Hz. For each tetrode, multiple spike sorting algorithms were run using the SpikeInterface python-based framework [34–36]. The spike sorters used were Tridesclous, SpikingCircus, Klusta, HerdingSpikes, Ironclust, and MountainSort4. The default parameters included in SpikeInterface per spike sorter were used. Agreement between the spike sorters was computed, and only putative neurons that were detected by at least two spike sorters were considered. When a tetrode didn’t have any consensus, the detections of the MountainSort4 spike sorter were used given that this spike sorter had better scores according to the SpikeForest [37] metrics and our own examinations. After the putative neurons were detected, an experienced user curated manually the detections by visual inspection using the Phy interface and labeled them as either good, multiunit activity, or noise.

### Spike waveform extraction

Sampled at 30 kHz, the prelimbic cortex tetrode channels were loaded and then filtered using a third-order Butterworth bandpass filter with a frequency range of 300–600 Hz. Neurons labeled as “good” were used for further analysis. A preliminary waveform per neuron was defined with an 82-sample window with 40 samples before and 41 samples after the spike timestamp (ST). For each neuron, STs were randomly permuted and a total of 2000 were selected. The permutation was done to avoid bias when selecting waveforms. These 2000 waveforms were averaged to obtain the mean spike waveform per neuron. Since tetrodes were used to record neuronal activity during the task and sleep, there were four average waveforms to choose from. The one with the highest peak amplitude was chosen. For each neuron, all STs were stored. Further, each neuron was assigned a unique ID. This process was performed for all neurons of all rats.

### Neuron classification

The average neuron waveforms calculated from rats 1, 2, 6, and 9 were categorized as “Vehicle” and stored in a 139 (t1) × 82 matrix, with 139 being the total number of putative neurons and 82 being the number of samples as mentioned before. Similarly, “RGS14” data from rats 3, 4, 7, and 8 was stored as a 353 (t2) × 82 matrix. Variables t1 and t2 were the total counts of neurons for the respective treatments. The bz_CellClassification.m script from Buzsaki lab’s GitHub was used to compute the trough-to-peak delay time and the spike width for the spike waveform of each putative neuron. The function ClusterPointsBoundaryOutBW.m of the same repository was modified to incorporate visualization of interneuron and pyramidal data for both treatments. Neurons were then visualized in a 2D plane with *x* and *y* axes as trough-to-peak delay and spike width, respectively. The set of coordinates of all putative neurons were fed into a GMM with two components in order to find the centroids of the clusters of pyramidal cells and interneurons. The cluster which contained spikes with high values of spike width and trough-to-peak delay was labeled as the pyramidal neurons cluster, while the remaining one was labeled as the interneurons cluster. For both clusters, a threshold of mean +/−2 *SD* with respect to their centroids was used to filter out the outliers. Next, the firing rates of the remaining neurons were calculated as is described in the following paragraph and those with extreme firing rate values were reinspected in the Phy interface by another experimenter to potentially discard remaining false positives. After this procedure, there were a total of 101 pyramidal neurons for the RGS14 treatment and 57 for Vehicle. The total number of interneurons were 18 for RGS14 and 7 for Vehicle. In the following analyses, only pyramidal neurons were used given the low number of interneurons detected.

### Cortical activity during ripples

Using the spike timestamps during NonREM sleep, the cortical pyramidal neuron response to hippocampal ripples was computed in a 2-s window defined around the peak of each ripple. For each ripple, the activity of all pyramidal neurons detected during that day was extracted. The spike timestamps in the 2-s window were normalized to vary from –1 to 1 s, where 0 was the ripple peak. After concatenating the normalized timestamps across all ripple-centered windows per neuron, they were binned in 10-ms bins and the number of spikes was determined for each bin. Hence, for each neuron, a [1 × 200] column vector was obtained. This vector was then z-normalized. The final z-scored vector was found by averaging the z-scored vector over all neurons. To reduce high-frequency jitter without obscuring the overall time course of the response, the data were smoothed twice using the MATLAB’s smooth function with its default five-bin moving average. To visualize the activity of prelimbic cortex pyramidal neurons around the ripple, the firing activity of a neuron was determined from a 50-ms window around the peak of the ripple (–20 to +30 ms) by quantifying the number of spikes. After compiling the firing activity around the ripple peak for each neuron the values were sorted in an ascending order and was visualized using the MATLAB imagesc function.

### Analysis by event, post-trial period or study day

For figures, we state if the analysis was done per event, per post-trial period or per study day. For general characteristics (e.g. durations, granger analysis), analysis was done per event including all events from all conditions and animals. Since the experiments were all run counterbalanced and all animals had roughly the same rate of events, the event and other types of analysis always have roughly equal contribution from each animal and results are not driven by one. For the sequence and co-occurrence analysis with other oscillations, we computed these for each post-trial period individually but only including post-trial periods that had at least 3 min of NonREM sleep. To compensate that each post-trial period would have different amounts of sleep, we normalized by the total count of the events in that post-trial period. For analyses with insufficient events per post-trial period (e.g. the fraction of C3-multiplets and spindle-locked ripples), data were aggregated across each study day. The specific analysis applied to each figure is detailed in its respective legend.

## Results

### Identifying ripple subtypes with principal component analysis

To uncover different ripple subtypes, various ripple features were computed from every ripple detected during NonREM sleep from its raw trace in 7-h recordings (430 min) with different conditions (Figure 1A, from 3 vehicle animals each 4 × 7-h recordings, for explanation of conditions, see Materials and Methods section, all conditions included), and a PCA was applied to the extracted features (Figure 1B and C). A Gaussian mixture model (GMM) clustering algorithm was applied on the three PCA dimensions, which accounted for most of the variance of the data (~90%). The number of clusters was determined using a consensus of Akaike and Bayesian information criteria (AIC, BIC) and the Calinski–Harabasz index, which indicated that four clusters balanced model fit and complexity. Clustering yielded four groups, but the fourth cluster contained only 378 events, compared with 28 857 (C1), 8525 (C2) and 11 039 (C3) events in the other cluster groups (Figure 1D; Figure S1A, 4A). Because of its extremely low representation, this cluster was treated as noise and excluded from subsequent analyses. Of note, clusters represent groupings in a continuous dataspace with no sharp boundaries between the different types, similar as described for individual theta cycles (Video S1 & Video S2) [38]. Figure 1E shows both individual examples as well as median values for the remaining three clusters. Interestingly, ripples from cluster 2 (C2) were distinct from the other two clusters as they lacked a slow frequency deflection in the signal. This deflection in C1 and C3 ripples may reflect the volume-conducted sharp-wave component originating in other hippocampal layers. C2 ripples also differed from the other clusters exhibiting a small rise after the initial deflection in the prelimbic cortex right before the ripple event (Figure 1E right).

For each recording period the fraction of ripples corresponding to each cluster was calculated, Cluster 1 was the largest cluster with 60% of ripples in contrast to cluster 2 and 3 with each 20% (Figure 1F, Figure S1, ripple rates M ± SEM: C1 = 17.8 ± 0.6, C2 = 5.4 ± 0.2, C3 = 6.8 ± 0.37 each per min). These findings were reproduced on the individual animal level, across the study day (Figure S1B-E). Interestingly, computing ripple characteristics revealed that ripples in cluster 2 were, on average smaller, slower and shorter than those in the other clusters, with the difference in size most pronounced in the area under the curve measure (AUC, Figure 1Fv). These ripples were also less phase-locked to the cortical slow oscillation (Figure 1F). In contrast, cluster 3 ripples were, on average, larger, faster, longer, and exhibited greater overall magnitude, as indicated by the AUC (Figure 1F, durations M ± *SD*: C1 = 0.044 ± 0.01, C2 = 0.041 ± 0.008, C3 = 0.08 ± 0.04 each in s, amplitudes M ± *SD*: C1 = 46.6 ± 18.85, C2 = 37.0 ± 14.7, C3 = 55.5 ± 19.7). They were as phased-locked to the slow oscillation as cluster 1 but occurred earlier in the cortical slow oscillation phase.

Next, we analyzed the activity of neurons in the prelimbic cortex around ripples (Figure 1G). Cluster 1 ripples had a higher average cortical, neuronal response (Figure 1G i). More neurons were C1-ripple-responsive (Figure 1G ii), and these neurons ramped up their activity more before the ripples than seen in other clusters (Figure 1G iii). In contrast, ripples in C3 came with a higher neuronal response after ripples.

We validated our PCA approach by also running a clustering of events extracted from the ripple-filtered (100–300 Hz) traces (in contrast to the raw trace). Extracted ripple types had the same basic characteristics (Figure S1G).

In sum, by applying a PCA to extracted ripple features, three distinct ripple types could be identified. The medium-sized ripple (here C1, terminology based on AUC) as well as the small-sized (here C2) and large-sized (here C3) ripple type. C2 and C3 ripples were less common than C1 ripples. Interestingly, while C2 and C3 ripples resulted in less cortical neuronal firing than C1 ripples, the response was sustained longer after C3 ripples.

### Hippocampal-cortical oscillation interactions during ripple types

Hippocampal ripples are known to interact with cortical regions, but the type of interaction may be specific to ripple types. Thus, we next analysed interactions with detectable events of slower sleep oscillations i.e., spindles and delta waves.

**Figure 2:**
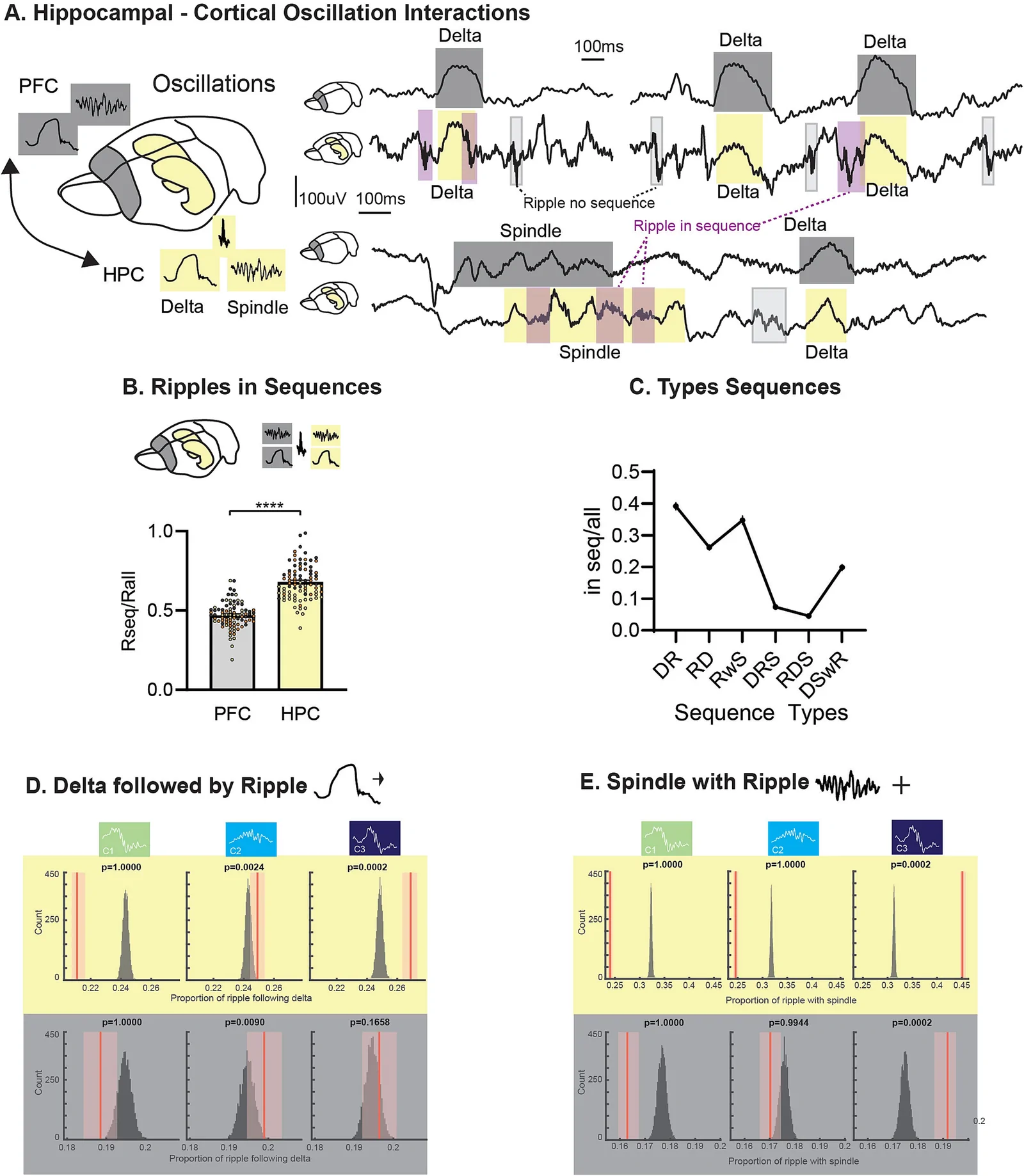
Hippocampal–cortical interactions: (A) Delta and spindle can occur in the prefrontal cortex (PFC) and hippocampus (HPC). (B) Fraction of ripples occurring in a sequence with a cortical (left) or hippocampal (right) oscillation (incl post-trial periods *t*_168_ = 9.17 *p* < .0001). (C) Fraction of ripples occurring in specific sequence types (DR, delta followed by ripple; RD, ripple followed by delta; RwS, ripple co-occurring with spindle; DRS, delta–ripple–spindle sequence; RDS, ripple–delta–spindle sequence; DSwR, delta followed by spindle co-occurring with ripple). Permutation analysis for occurrence of ripples after delta’s (D) and during spindles (E) ^****^p < .0001. All error bars are SEM.

Ripples are known to be coupled to sleep oscillations such as delta waves [18, 39] and spindles [4, 18, 39–42]. Thus, we detected delta and spindle oscillations in both the prefrontal cortex and hippocampus and quantified the fraction of ripples that occurred in sequence with either a cortical or hippocampal oscillation (delta wave or spindle, before, during, after, Figure 2A). Of note, we detected delta deflections in the signal that can represent both slow wave activity as well as slow oscillations with their on and off-states [43]. Thus, the delta detections here are different then the slow oscillation phase analysis (Figure 1Fv), which measures a continuous oscillatory signal. Ripples in general were more likely to be in a sequence with a hippocampal delta or spindle oscillation than a cortical one (Figure 2B). We split the sequences in types of events, focused on the most common sequences (Figure 2C, percentage of ripples occurring in sequence: M ± SEM, DR = delta-ripple 0.39 ± 0.01, RD = ripple-delta 0.26 ± 0.008, RwS = ripple with a spindle 0.35 ± 0.013, DRS = delta-ripple-spindle 0.07 ± 0.005, RDS = ripple-delta-spindle 0.046 ± 0.004, DSwR = delta-spindle with a ripple 0.198 ± 0.009, see also Figure S2A): Delta followed by a ripple (DR) and ripples occurring during spindles (RwS) [4, 14]. Cluster 2 ripples were more likely to occur following both hippocampal and cortical deltas (permutation test; *p* = .0024, 0.0090, respectively, Figure 2D). In contrast, cluster 3 ripples occurred more than other ripple types after hippocampal deltas or during both hippocampal and cortical spindles (permutation test; *p* = .0002, 0.0002, 0.0002, respectively, Figure 2D, E).

**Figure 3:**
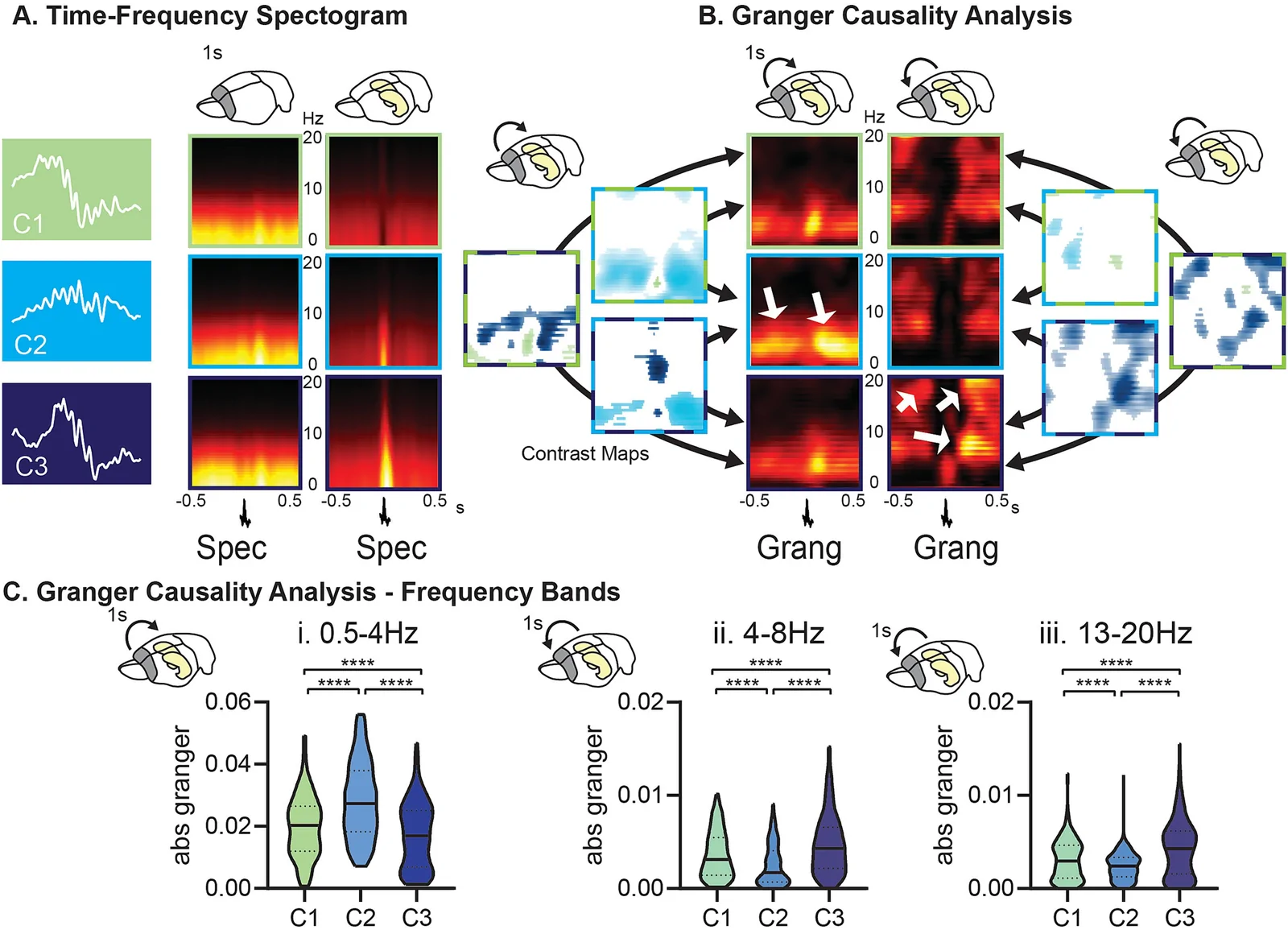
Time–frequency and Granger causality analyses: (A) Spectrograms (Spec) and (B) Granger time–frequency analysis (Granger) for prelimbic cortex and hippocampus in lower (0–20 Hz, 1-s window around ripple, left and right cluster-corrected significant differences between ripple clusters indicated by the arrows) frequency ranges. Clusters differ in the hippocampal sharp-wave (0–4 Hz) power, as well as Granger PFC → HPC in the delta and Granger HPC → PFC in the theta (4–8 Hz) and spindle/beta (~20 Hz) range (indicated by arrows). Statistical contrast maps indicate higher values for Cluster 1 (C1), Cluster 2 (C2), or Cluster 3 (C3) respectively (pixel-based, nonparametric permutation test corrected for multiple comparisons). (C) Extracted Granger values per event for specific frequency bands (i) 0.5–4 Hz (PFC → HPC *F*_2,2711_ = 1302 *p* < .0001, Tukey post hoc) and (ii) 4–8 Hz (HPC → PFC *F*_2,3050_ = 604 *p* < .0001, Tukey post hoc), and iii 13–20 Hz (HPC → PFC *F*_2,5084_ = 391 *p* < .0001, Tukey post hoc) ^*^p < .05, ^**^p < .01, ^***^p < .001, ^****^p < .0001. All error bars are SEM.

Lastly, we computed the spectrograms around the ripples for each cluster and performed time-frequency Granger causality analysis (Figure 3A and B). We identified significant changes in connectivity with a pixel-based, nonparametric permutation test corrected for multiple comparisons. This analysis was performed on a subset of 2000 ripples per cluster, selecting those with amplitudes closest to each cluster’s median, ensuring the same number of events for each cluster (see Figure S7 for control). Cluster 2 was distinguished by higher Granger values from the prefrontal cortex (PFC) to the hippocampus (HPC) PFC → HPC in the upper delta range, both before and after the ripple, compared to the other clusters. In contrast, C3 ripples showed higher HPC → PFC Granger values before and after the ripple in the theta and spindle/beta range in comparison to both other clusters. These patterns were confirmed when extracting the granger values for these frequency bands and performing ANOVAs (Figure 3C). C3 ripples also came with higher connectivity for PFC → HPC in 100–300 Hz (Figure S2).

In sum, C2 ripples occurred more frequently after delta events, while C3 ripples occurred more frequently after hippocampal delta events and during spindles. Finally, C2 ripples showed distinct PFC → HPC connectivity in the upper delta range, whereas C3 ripples showed distinct HPC → PFC connectivity in the theta and spindle ranges.

### Ripple Multiplets increase with complex learning and foster a hippocampal-cortical dialogue

**Figure 4:**
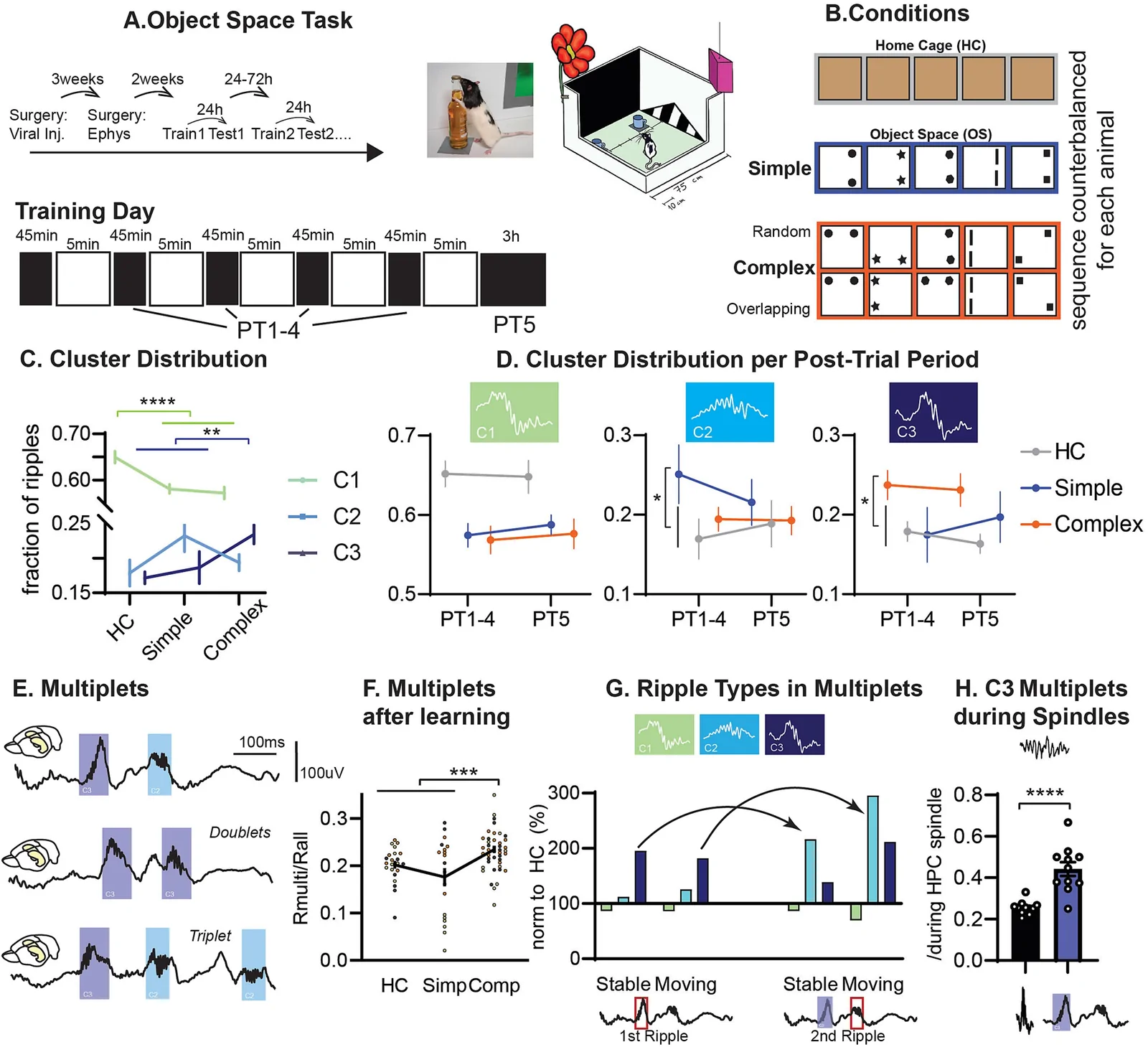
Learning specific effects—multiplets and spindles. (A) Object Space Task allows animals to explore objects in an open field. (B) Home cage (HC) was compared to simple and complex learning conditions. (C) Fraction of ripples per PT period classified as C1, C2, or C3 split for different behavioral conditions (including post-trial period two-way ANOVA condition *F*_2, 246_ = 0 *p* > .99, cluster type *F*_2, 246_ = 625.5 *p* < .0001, interaction *F*_4, 246_ = 7.3 *p* < .0001) (D) Same as (C) but split for post-trial (PT) period. (E) Example of ripple multiplets, here with C3 and C2 ripples. (F) Fraction of ripples that were part of multiplets split for condition (including post-trial periods *F*_2,82_ = 7.6 *p* = .0009). (G) Ripple cluster type that was the first ripple of the multiplet (left). Right: Taking C3 ripple events and testing which type is the second ripple. Per-event analysis, each normalized to home cage. (H) Fraction of C3-Multiplets and all ripples occurring during spindles (per study day, *t*_21_ = 5.6 *p* < .0001) ^*^p < .05, ^**^p < .01, ^***^p < .001, ^****^p < .0001. All error bars are SEM.

After establishing the general properties and cross-brain coupling signatures of each ripple type, we next investigated whether different learning experiences were associated with specific ripple sub-types. For this dataset, animals were trained in the OST (Figure 4A) during which they have the opportunity to explore different object-pairs in an open-field environment. As shown previously, the task tests semantic-like memory and animals express the cumulative memory—the statistical distribution of the objects—over trials [4,14,23,44]. We compared a home cage control (remaining in recording box) to different Object Space Task conditions (Figure 4B): The simple learning condition—same object locations for five trials—and the complex learning conditions—different object locations for five trials. Complex learning consisted of two subconditions: Random (all locations used the same number of times) and Overlapping (one location always has an object, second location changed from trial to trial); however, we have previously shown that these two conditions result in the same consolidation signatures during sleep and present the results combined here for a more detailed discussion and behavioral results, please see Navarro-Lobato et al [4]. Interestingly, after both simple and complex learning fewer C1 ripples were observed (ripple rates M ± SEM: HC = 19.93 ± 1.3, Simple = 14.3 ± 1.5, Complex = 18.8 ± 0.94 each per min). In contrast, C3 ripples’ occurrence increased in complex learning (Figure 4C ripple rates M ± SEM: HC = 5.5 ± 0.5, Simple = 5.6 ± 1.1, Complex = 8.2 ± 0.6 each per min). Furthermore, when separating for sleep between training trials (post-trial 1–4) and after training (post-trial 5), we observed a stronger effect in PT1–4 than PT5 with more C2 ripples after simple and more C3 ripples after complex learning (Figure 4D).

Ripples are known to occur in bursts, with multiple ripples in close succession, i.e. doublets or multiplets. Thus, next, we tested how many ripples occurred in multiplets and which ripple types contributed to multiplets (Figure 4E). After complex learning the fraction of ripples part of multiplets increased (Figure 4F, fraction of ripples HC 0.20 ± 0.008, Simple 0.17 ± 0.019, Complex 0.23 ± 0.008). Interestingly, in comparison to home cage, learning before sleep—both simple and complex learning—shifted which types of ripples were part of multiplets. After learning, the first ripple was more likely to be a C3 ripple and these C3 ripples were then more likely to be followed by a C2 ripple or another C3 ripple (Figure 4G, Figure S2E for latencies). Finally, it has been proposed that multiplets occur during spindles [45], thus we compared the fraction of C3-led multiplets that occurred during spindles to general ripple–spindle coupling. C3-led multiplets were more likely to occur during a spindle than other ripples (Figure 4H).

In summary, simple learning, an object location that was reinforced from trial to trial, was associated with a shift characterized by a decrease in C1 ripples and an increase in C2 ripples, whereas complex learning, with changing object locations from trial to trial, resulted in a higher occurrence of C3 ripples. Additionally, complex learning induced more ripple multiplets, which alternated between C3 and C2 ripple events, and occurred during spindles.

### Delta and spindle cross-brain coupling and learning

**Figure 5:**
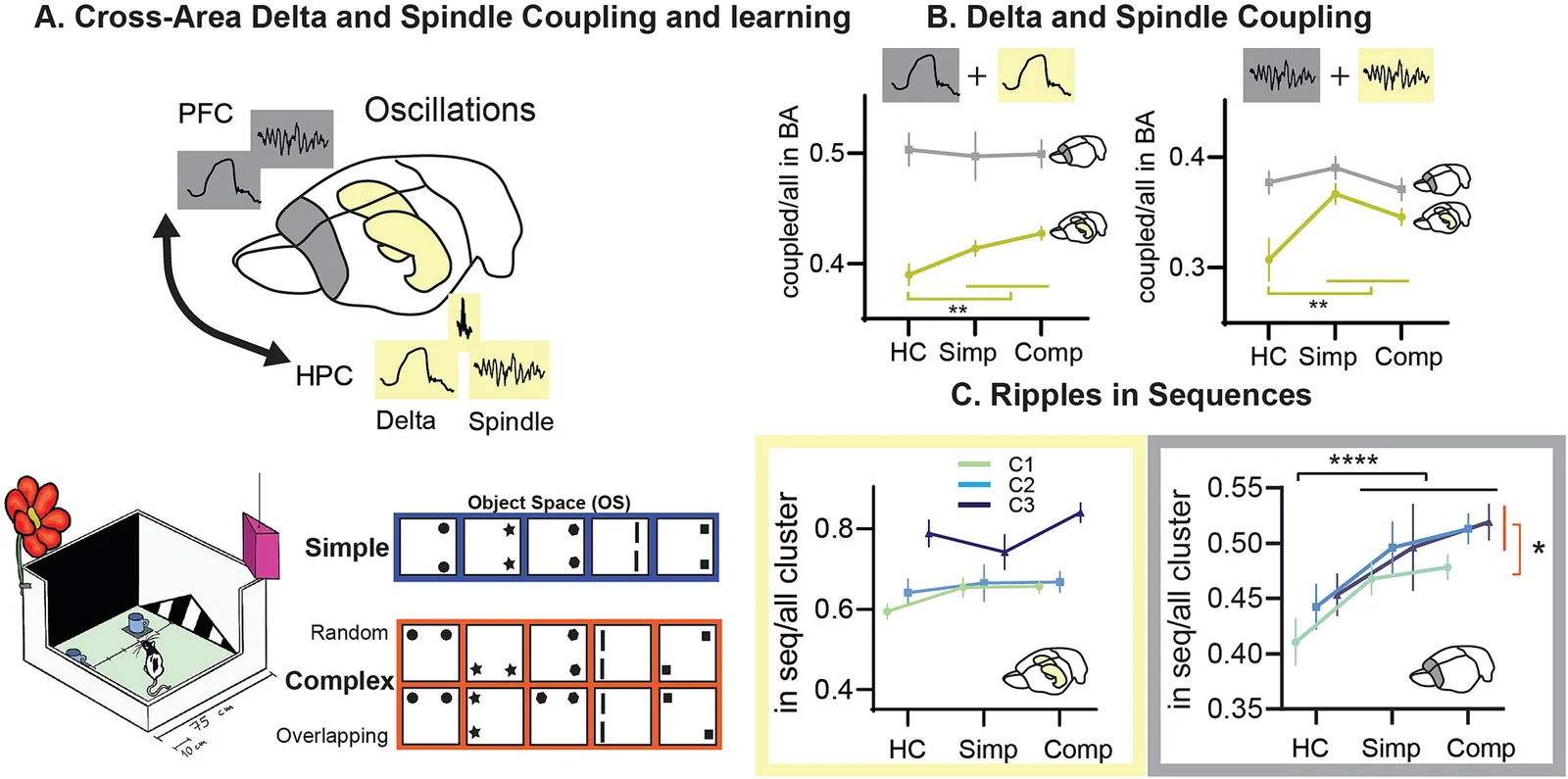
Learning specific effects—sleep oscillations (A) Home cage (HC) was compared to simple and complex learning conditions. (B) Fraction of cortical and hippocampal delta (including post-trial period left, two-way ANOVA condition *F*_2, 166_ = 0.9 *p* = .4, brain area *F*_1, 166_ = 70.6 *p* < .0001, interaction *F*_4, 166_ = 1.4 *p* = .25) and spindle (including post-trial period right, two-way ANOVA condition *F*_2, 166_ = 4.2 *p* = .02, brain area *F*_1, 166_ = 17.5 *p* < .0001, interaction *F*_4, 166_ = 2.3 *p* = .1) that occurred coupled to a corresponding event (i.e. also delta or spindle) in the other brain area split for behavioral condition (C) Fraction of ripples occurring in a sequence with a cortical (including post-trial period left, two-way ANOVA condition *F*_2, 246_ = 9.4 *p* = .0001, cluster type *F*_2, 246_ = 3.0 *p* = .0553, interaction *F*_4, 246_ = 0.03 *p* = .99) or hippocampal (including post-trial period right, two-way ANOVA condition *F*_2, 246_ = 2.4 *p* = .09, cluster-type *F*_2, 246_ = 23.3 *p* < .0001, interaction *F*_4, 246_ = 1.1 *p* = .35) oscillation split for cluster and condition**.** These sequences include ripples occurring before or after delta waves, as well as during spindles. ^*^p < .05, ^**^p < .01, ^***^p < .001, ^****^p < .0001. All error bars are SEM.

Next, we investigated the delta and spindle cross-brain coupling (i.e. delta–delta and spindle–spindle) and how it related to our learning conditions (Figure 5A). We saw that hippocampal delta and spindle events were more coupled to their cortical counterparts after learning in comparison to home cage (both simple and complex learning, Figure 5B). Interestingly, this led to more ripples being part of cortical sequences, while there was no such effect for hippocampal sequences (Figure 5C, Figure S2C and D, these sequences include ripples occurring before or after delta waves, as well as during spindles).

In summary, learning enhanced cortical–hippocampal coupling (both delta–delta and spindle–spindle), leading to an increase of ripples occurring in cortical sequences.

### Increasing plasticity in the prefrontal cortex changes proportion of ripple subtypes

**Figure 6:**
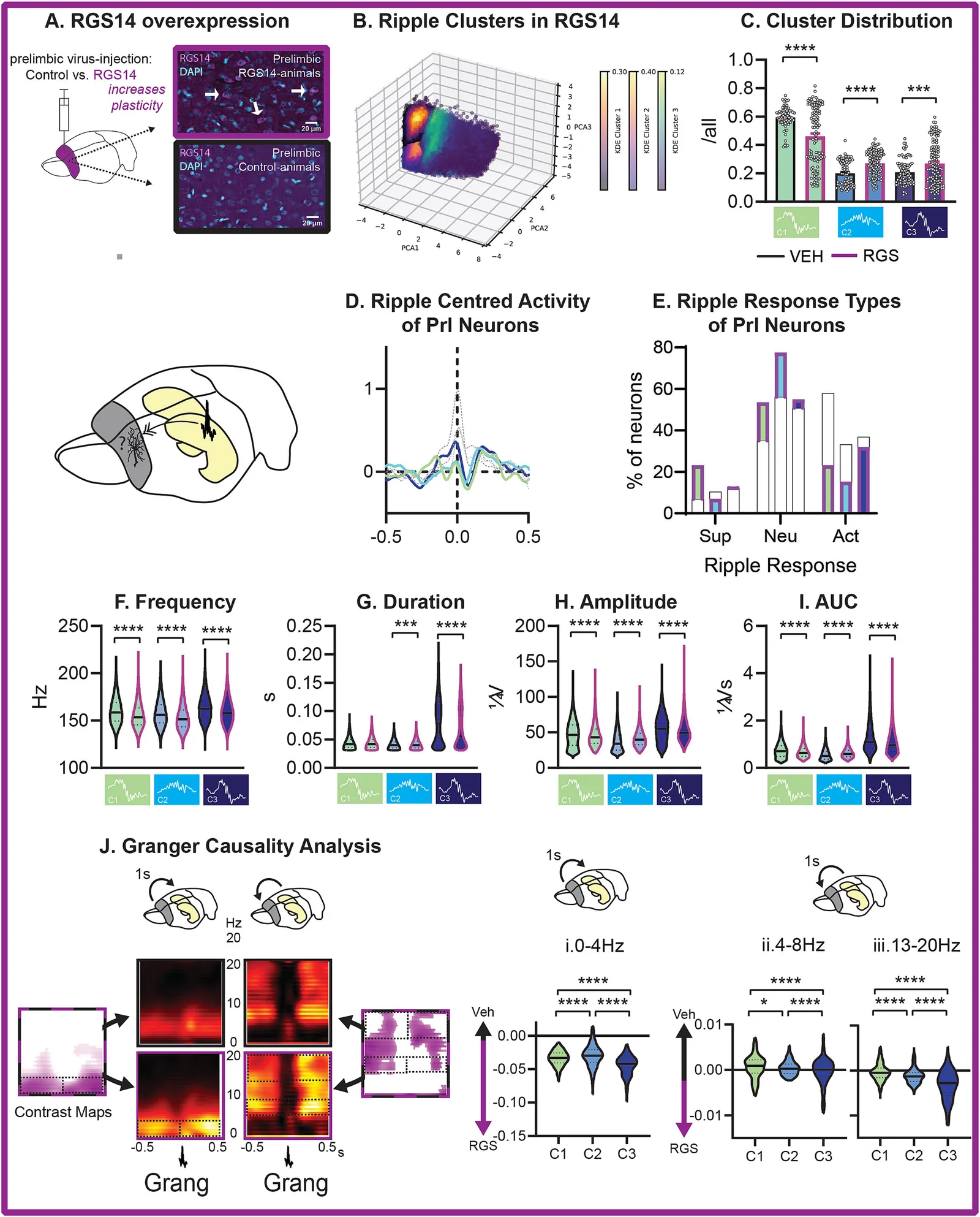
Increasing cortical plasticity: (A) RGS14 was injected into the prelimbic cortex leading to increased plasticity. (B) In RGS14, animals the same four clusters were found. (C) Fraction of ripples per cluster (including post-trial period Welch test, each *t* > 3.6 *p* < .0001), (D) Ripple centred responses of prelimbic neurons. (E) Percentage of ripple-active, neutral, or suppressed neurons computed across the full sample. (F) Ripple frequency (RGS vs Veh, C1 Mann–Whitney *U* = 424642034 *p* < .0001, C2 Mann–Whitney *U* = 96 920 700 *p* < .0001, C3 Mann–Whitney *U* = 111 598 030 *p* < .0001), (G) duration (RGS vs Veh, C1 Mann–Whitney *U* = 527 647 875 *p* = .87, C2 Mann–Whitney *U* = 117 003 878 *p* = .0002, C3 Mann–Whitney *U* = 115 250 218 *p* < .0001), (H) amplitude (RGS vs Veh, C1 Mann–Whitney *U* = 509 056 059 *p* < .0001, C2 Mann–Whitney *U* = 91 332 080 *p* < .0001, C3 Mann–Whitney *U* = 122 609 173 *p* < .0001), (I) area under the curve (AUC, RGS vs Veh, C1 Mann–Whitney *U* = 338 700 485 *p* < .0001, C2 Mann–Whitney *U* = 93 272 663 *p* < .0001, C3 Mann–Whitney *U* = 112 977 834 *p* < .0001), (F–I) including all events. (J) Granger causality analysis (event analysis on subset of 2000 events each) with Granger values extracted for each subtype for (i) 0–4 Hz (delta, ANOVA *F*_2,2711_ = 719.2 *p* < .0001, Tukey post hoc), (ii) 4–8 Hz (theta ANOVA *F*_2,3050_ = 53.8 *p* < .0001, Tukey post hoc), and (iii) 13–20 Hz (spindle/beta ANOVA *F*_2,5084_ = 699.1 *p* < .0001, Tukey post hoc). ^*^p < .05, ^**^p < .01, ^***^p < .001, ^****^p < .0001.

To test whether the occurrence of ripple types is under top-down control of the cortex, we artificially increased plasticity in the prelimbic cortex via the overexpression of an established plasticity enhancer called regulator of G protein signaling 14 of 414 amino acids (RGS14, Figure 6A, Figure S8) [25,46]. RGS14 overexpression is known to lead to increased BNDF expression and dendritic branching in the targeted area [25,46], thereby enhancing local plasticity. We have previously shown that this cortical manipulation leads to *overlearning*, in which all experiences—including home cage control—induce consolidation signatures. Furthermore, we showed that these animals have enhanced one-trial learning, which leads to more interference on semantic-like memory (Figure S6) [4]. Using the PCA loadings from the vehicle animals, we identified the same ripple clusters in RGS14 animals (Figure 6B, Video S3, Figure S4 and S5). RGS14 animals had a higher fraction of C2 and C3 ripples and fewer C1 ripples (Figure 6C). The same shift was visible in absolute event rates (Figure S5B and for individual animals Figure S1D and E). We previously reported [4] that RGS14 manipulation led to both lower neuronal firing rates in the prelimbic cortex and decreased ripple-related increases in cortical neuron firing rates. Now we show that this held across all ripple types, with lower average ripple-triggered responses, as well as fewer neurons classified as ripple active (Figure 6D and E). All three types of ripples had slower frequencies in RGS14 animals than in vehicle animals. Only C3 ripples were significantly shorter in duration, while the other ripple types had similar durations between groups. Interestingly, while RGS14 animals showed smaller amplitudes and AUC in C1 and C3 ripples, C2 ripples were larger and had higher AUC (Figure 6H and I). Across all ripples, granger causality values in RGS14 animals were generally higher in delta frequencies (PFC → HPC) and in theta and spindle/beta frequencies (HPC → PFC), as also reported in Navarro-Lobato et al [4]. When investigating subtypes separately, we could show that the effect seen when including all ripples, was mainly driven by differences in C3 ripples, while other ripples subtypes showed smaller differences in comparison to vehicle (Figure 6J). Interestingly, especially in the PFC → HPC delta range, C2 ripples were even less affected than C1.

In sum, increased plasticity in the prelimbic cortex, led to more C2 and C3 ripples and increased cortical–hippocampal coupling during the home cage condition, consistent with the previously described *overlearning* phenomenon [4]. Interestingly, C3 ripples showed larger differences from vehicle than the other subtypes when the cortex was manipulated.

## Discussion

In the present study, we utilized an unsupervised analysis framework to isolate distinct subtypes of NonREM sharp-wave ripples (SWRs) based on their intrinsic physiological features. Consistent with prior literature, this approach segregated SWRs into a dominant medium-sized cluster (C1; ~60% of events) and two structurally distinct variants: small (C2) and large (C3) ripples, each comprising approximately 20% of the total population.

The central novelty of our findings lies in mapping these physiological variants onto specific behavioral demands and directional routing states during systems consolidation. Specifically, small-sized (C2) ripples—which preferentially follow cortical delta waves and exhibit enhanced top–down, cortical–hippocampal connectivity—were predominantly upregulated following simple learning, where object locations remained stable across trials. This dynamic suggests that when spatial rules are stable, cortical down-to-up state transitions may act to gently query the hippocampus to reinforce an existing representation.

Conversely, large-sized (C3) ripples—which are tightly coupled with thalamocortical spindles and drive bottom–up, hippocampal–cortical connectivity—dominated the sleep period following complex learning, where object locations were continuously updated. Because adapting to shifting environments requires updating cortical representations, this spindle-nested, bottom–up connectivity may reflect the active transfer of newly formed hippocampal sequences to distributed neocortical networks.

Perhaps most intriguingly, complex learning also induced a shift from isolated SWRs to alternating bursts of C2 and C3 events. Rather than simple unidirectional signalling, this alternating architecture could imply an iterative “query-and-return” loop—a sustained, dynamic dialogue between the hippocampus and cortex.

### Comparison to other ripple types

Hippocampal SWRs are increasingly recognized not as a monolithic event, but as a highly heterogeneous ensemble of events. The timing and structure of SWRs are heavily biased by varying inputs from the dentate gyrus and entorhinal cortex, while cortical slow oscillations and spindles can advance or delay ripple occurrence, leading to irregular firing patterns [7, 10]. Furthermore, unsupervised analyses indicate that ripple-related firing varies across deep and superficial CA1 pyramidal cell sublayers, reflecting differences in input sources and local inhibition gradients [19,21].

In our dataset, we identified three ripple types. Notably, these variants were derived from a continuous data pace without sharp boundaries, aligning with recent perspectives that SWRs exist on a physiological spectrum determined by the instantaneous balance of circuit inputs [19, 38]. Despite this continuity, our identified extremes exhibited robust variations in classic features such as amplitude, frequency, and duration, alongside critical differences in cortical slow oscillation phase-locking and the presence of a sharp-wave deflection.

These distinctive features enabled us to compare our identified ripple types with previously described classifications.

Specifically, our small-sized (C2) ripples lacked an accompanying sharp-wave deflection. This profile is highly consistent with the “sharp-wave-less” ripple subtypes described previously by Ramirez-Villegas et al. [20] the “region C” subtype in [19] and the variants detailed by Aleman-Zapata et al. [13]. Because a classic sharp-wave deflection reflects a massive, synchronous discharge from CA3 Schaffer collaterals into the CA1 stratum radiatum [47], the absence of this deflection suggests that C2 ripples are not solely driven by CA3. Instead, they likely originate from alternative excitatory sources. For instance, CA2 neurons, which ramp up their activity prior to SWRs, have been proposed as potent initiators of ripple events [48]. Simultaneously, medial entorhinal afferents terminating in the stratum lacunosum-moleculare (SLM) provide a powerful alternative drive to the CA1 network [7,10,21].

This entorhinal-dominant mechanism is strongly supported by our previous analyses and findings, where we found that events lacking a sharp-wave deflection—akin to our C2 class—exhibited a distinctive current-source density (CSD) profile characterized by pronounced sinks in the SLM and attenuated sources in the stratum radiatum [13]. Crucially, this localized anatomical drive contextualizes our network-level findings: our Granger causality and phase-coupling analyses revealed that these C2 ripples are preceded by local delta waves—lacking the down state phase characteristic of global slow oscillations [10, 49, 50]—and coordinated by prelimbic cortical activity. This suggests that top–down prefrontal pathways—likely acting via the entorhinal cortex or thalamic nucleus reuniens to target the SLM [51]—can effectively bypass the classic CA3 pathway to directly “query” the CA1 network during simple learning states. Behaviorally, C2 ripples were most prevalent during simple learning in the stable condition of the Object Space task, where the object locations are reinforced from trial to trial. Under these circumstances, where simple associations are likely to be consolidated, cortical information may be assimilated rapidly, and small-sized ripples were the predominant subtype observed. Furthermore, C2 ripples were less affected by the cortical plasticity manipulation and became more numerous, consistent with a potential cortical influence on their generation.

In stark contrast to the sharp-wave-less C2 events, large-sized (C3) ripples exhibit robust physiological features indicative of powerful, synchronous CA3 Schaffer collateral drive. Classically, the sharp-wave component of an SWR manifests as a prominent negative deflection in the CA1 stratum radiatum, reflecting this massive CA3 input [47]. Recent large-scale recordings demonstrate that the magnitude of this sharp-wave sink scales directly with ripple power, frequency, duration, and the synchronized recruitment of extended CA1 and CA3 neuronal assemblies [9, 10, 52]. Our identified C3 events align with this “classical” high-drive profile: they exhibit the largest amplitude, fastest intraripple frequency, and longest duration of all identified classes, coupled with strong spectral power in both the ripple and sharp-wave bands.

These C3 events closely resemble the prolonged SWRs typically observed during post-learning wakefulness [12] and sleep [13, 14], as well as the “classical ripples” (subtypes 2–3) detailed by Ramirez-Villegas, Logothetis, and Besserve [20]. Functionally, these high-power, prolonged ripples may be important for complex memory consolidation. Extended SWRs are known to accompany large-scale hippocampal–cortical reactivations, and optogenetically prolonging SWR duration has been shown to actively rescue and boost weak memory traces [11].

Crucially, our network-level analyses demonstrate that C3 ripples serve as a possible primary vehicle for this cortical-updating process. Following complex learning, C3 events triggered prolonged cortical firing and were characterized by robust, bottom-up hippocampo-prefrontal coupling. This connectivity was heavily concentrated in the theta and spindle/beta bands, occurring precisely during the execution of hippocampal spindles. The resulting Granger causality “hotspots” align with cortical spectrograms observed during targeted memory reactivation paradigms [53] potentially suggesting that C3 actively update newly formed spatial representations to the neocortex via coordinated spindle oscillations.

Finally, our cortical plasticity manipulation offers potential support for this regulated inter-regional dialogue. The intervention preferentially impacted this bottom-up pathway, disproportionately targeting C3 ripples and attenuating their distinct connectivity signatures. These findings suggest that C3 ripples are not simply isolated hippocampal events, but rather integrated components of a distributed, bidirectional cortico-hippocampal network.

The final cluster, medium-sized ripples (C1), occupied an intermediate physiological parameter space, exhibiting frequencies and amplitudes that bridged the C2 and C3 extremes. While their durations occasionally overlapped with the shorter C2 variants, C1 events were primarily distinguished by their behavioral context: they constituted the vast majority (~60%) of default SWRs during periods of basal activity and non-learning sleep.

This default prevalence aligns with theoretical models proposing that a subset of SWRs are generated stochastically through rigid anatomical pathways, serving to maintain preconfigured, backbone network structures rather than updating novel information described by Hall and Wang [54]. Functionally, these baseline events likely target the “rigid” subpopulation of CA1 pyramidal neurons described by Grosmark and Buzsaki [55]—cells whose firing dynamics remain strictly stable across pre- and post-learning sleep periods. This “housekeeping” role is further supported by perturbation studies showing that disrupting ripples during non-learning periods fails to trigger a homeostatic upregulation [56]. Because C1 ripples may simply maintain routine, familiar representations, a homeostatic rebound is unnecessary. Accordingly, our data demonstrates that this default C1 population is downregulated following active learning, yielding the network behavior to shift towards more specialized C2 and C3 variants.

Interestingly, despite their baseline status, we found that C1 ripples elicited the highest-magnitude neuronal responses in the cortex. However, rather than reflecting efficient information transfer, this broad activation likely represents an unrefined, default state of network excitability. In contrast, the learning-induced shift away from C1 events resulted in a distinctly sparser cortical response. This fits with principles of memory consolidation, wherein learning refines broad, generalized network excitability into sparse, highly specific, and computationally efficient neocortical engrams [52, 57].

The physiological divergence of our identified SWRs maps remarkably well onto recent high-density laminar profiling studies, which categorize ripples into distinct Lacunosum Moleculare sink “LM^sink^” and Radiatum sink “Rad^sink^” motifs [58, 59]. These established profiles correspond to our small-sized (C2) and large-sized (C3) variants, respectively, and are known to recruit structurally distinct neuronal ensembles. Rad^sink^ (C3) events heavily integrate recent motifs of waking coactivity, combining both superficial and deep CA1 principal cells into dense, high-dimensional patterns capable of stable, hour-long reactivation [58]. In contrast, LM^sink^ (C2) events rely on core motifs of prior coactivity, engaging deep CA1 cells into sparser, lower-dimensional patterns that undergo a gradual “reactivation drift” to softly update pre-structured content [60].

These distinct ensemble dynamics integrate with our behavioral findings. The dense, high-dimensional reactivation driven by Rad^sink^/C3 ripples provides the necessary computational bandwidth for complex learning, where the network should continuously adapt to shifting spatial contingencies from trial to trial. Conversely, the sparser, core-motif reliance of LM^sink^/C2 ripples is ideally suited for simple learning, where stable spatial information is simply reinforced. While our findings strongly implicate these discrete functions, utilizing closed-loop targeted interventions to causally isolate these specific subtypes during consolidation remains a critical experiment for future research.

Because our unsupervised principal component space was derived from three distinct behavioral states (home-cage, simple learning, and complex learning), it inherently reflects the variance of these specific conditions. While incorporating a wider array of cognitive paradigms could potentially unveil further SWR subdivisions, the hypothesis that our identified subtypes are merely transient, task-dependent artifacts is unlikely. Crucially, all three ripple classes were reliably observed across all subjects and all behavioral conditions. Rather than strictly condition-dependent functional modes, these SWR subtypes likely represent canonical, highly conserved physiological motifs, the expression ratios of which dynamically shift to meet instantaneous cognitive demands.

In summary, our identified SWR variants align with established anatomical profiles and represent fundamentally distinct hippocampo-prefrontal computational states. By demonstrating that small (C2) and large (C3) ripples govern opposing directions of cortico-hippocampal communication and are distinctly recruited by simple versus complex learning demands, we provide a potential framework for understanding how heterogeneous SWRs route specific memory computations during sleep.

### Oscillation sequences depending on simple or complex learning

Beyond their isolated physiological features, our identified SWR subtypes exhibited dynamic shifts in how they participated within broader cortical and hippocampal oscillation sequences. A critical distinction in our network analysis is the definition of the “delta” activity under scrutinization. The delta deflections detected in our event sequences likely reflect transient, localized slow waves, distinct from the global, brain-wide up-and-down states typically captured by standard slow oscillation-phase analyses. Within this localized framework, ripples are known to reliably occur adjacent to delta waves [18, 39, 60] and tightly nested within or adjacent to discrete spindle events [18, 41, 42, 61, 62]. Building upon our previous work demonstrating that different behavioral experiences generate highly specific oscillation-event sequences [3, 4, 14], our current data allow us to map specific SWR physiological variants directly onto these task-dependent networks.

Building on this framework, our data reveal a dissociation in how simple versus complex cognitive demands recruit these specific oscillatory motifs. Following simple, reinforced learning, the network preferentially engages delta–C2 sequences. Because these C2 events are reliably preceded by local delta waves, this sequence supports a top–down, cortically driven mechanism designed to query and stabilize existing, well-learned spatial maps.

Conversely, complex learning—which requires the continuous updating of novel object locations—triggers a robust shift toward C3-mediated sequences. We previously established that general delta-ripple sequences, particularly those involving longer ripples, increase specifically following the complex conditions of the OST [4, 14]. Here, we refine this view by demonstrating that C3 ripples uniquely dominate these high-demand updating periods, occurring both within hippocampal delta-ripple sequences and tightly coupled to discrete spindles. Previous work has linked these delta-ripple sequences to prelimbic reactivations during complex rule learning [39]. Our network analyses provide a potential mechanism for this observation: C3 ripples drive intense bilateral communication, characterized by dominant bottom–up HPC → PFC connectivity in the theta and spindle/beta bands, alongside fast-frequency PFC → HPC feedback. This bidirectional, spindle-nested architecture is the hallmark of active memory updating, orchestrating the transfer of newly formed, continuously shifting spatial trajectories from the hippocampus to distributed neocortical storage sites.

The primary effect of both learning conditions was an augmentation of hippocampal–cortical coupling of delta and spindle oscillations. While this outcome is not unexpected [63], our study unveiled a novel finding: ripple subtypes inherently exhibited a default coupling with hippocampal delta and spindle events. Following learning, hippocampal delta and spindle activities became more synchronized with their cortical counterparts. Consequently, hippocampal ripples were more prone to occur in sequences alongside cortical delta and spindle oscillations, a pattern that may facilitate cortical memory network updating. Of note, with our current recording technique we cannot exclude that spindles and deltas detected in the hippocampus were not volume projected events from nearby cortices.

The augmented hippocampo-cortical coupling observed following learning is likely orchestrated by distributed thalamic networks. The anterior thalamic nucleus, an established pacemaker for synchronizing cortical slow oscillations and spindle activity [51, 64, 65], provides a highly plausible entrainment mechanism for this cross-regional dialogue. By locking hippocampal ripples into these widespread, thalamically mediated slow-frequency sequences, the network effectively opens temporal “windows of receptivity” across the cortex. Within these synchronized windows, the distinct SWR subtypes operate as specialized communication nodes: C2 ripples align with top–down cortical selection of existing hippocampal outputs, while C3 ripples drive the bottom–up reinforcement of novel information.

However, these functional inferences must be interpreted within the context of certain experimental limitations. First, while our sequence analyses rely on localized LFP features, standard in vivo recording techniques cannot definitively exclude the possibility that some delta and spindle events detected in the hippocampus reflect volume conduction from adjacent cortical tissues. Second, the associations reported here between SWR subtypes and cognitive demands are correlational. Because the simple and complex task conditions inherently differ in overall information load and updating demands, isolating the precise contribution of an individual ripple subtype from broader task-state effects remains challenging.

Direct, closed-loop manipulation of specific SWR subtypes will be required to causally separate these variables. Nevertheless, our current findings provide evidence that learning dynamically upregulates the cross-brain coupling of slower frequency rhythms, establishing a structured temporal scaffold that enables highly specialized, subtype-specific information transfer during ripples.

## Conclusion

By applying an unsupervised, data-driven framework to our in vivo recordings, we demonstrate that hippocampal SWRs are not monolithic events but comprise distinct physiological variants with highly specialized routing functions. While default, medium-sized (C1) ripples govern rigid, baseline network states, cognitive demands dynamically shift the brain toward targeted modes of inter-regional communication. Specifically, simple, reinforced learning preferentially upregulates small (C2) ripples, which act as top–down, delta-coupled queries to stabilize existing spatial maps. Conversely, complex learning—which requires the continuous updating of novel schemas—recruits large (C3) ripples to drive the bottom–up export of new memory traces via spindle-nested sequences.

Crucially, we show that learning actively enhances the cross-brain synchronization of these slow-frequency rhythms, forging a highly structured temporal scaffold. Supported by targeted cortical plasticity manipulations that specifically disrupt this bottom–up dialogue, our findings reveal a unified mechanism for systems consolidation: the brain resolves varying cognitive demands during sleep through a bidirectional, temporally precise cortico-hippocampal dialogue, dynamically routed by heterogeneous SWR subtypes.

## Supplementary Material

Supplementary_Material_zsag168

## Data Availability

All data are available at https://osf.io/7xw43/ . Code can be found on the following GitHub links: https://github.com/genzellab/RGS14 and https://github.com/genzellab/RGS14_clusters

## Preprint repositories

https://doi.org/10.1101/2025.03.19.644132
