# Supplementary material for: Deltas’ and spindles’ cross-area synchronization and ripple subtypes
Source: Sleep. 2026 Jun 19;49(9):zsag168. doi: 10.1093/sleep/zsag168 (PMC13553294; doi:10.1093/sleep/zsag168)
Supplement: Supplementary file 1 [file supplementary_material_zsag168.zip › Supplementary_Material_zsag168.docx]

**Deltas’ and Spindles’ Cross-Area Synchronization and Ripple Subtypes**

**– Supplementary Materials**

Adrian Aleman-Zapata^1^, Yixiao Zhang^1^, Pelin Özsezer^1^, Kopal Agarwal^1^, Abdelrahman Rayan^1#^, Irene Navarro-Lobato^1#^, Lisa Genzel^1#^*

^1^Donders Institute for Brain, Cognition and Behaviour, Radboud University, Postbus 9010 6500GL Nijmegen, The Netherlands.

#contributed equally

*Corresponding author: Lisa.Genzel@donders.ru.nl

Video 1: Rotating 3D view of ripples in PCA space with and without clusters.

Video 2: Rotating 3D view of ripples in PCA space split for clusters

Video 3: Rotating 3D view of ripples in PCA space split for clusters in RGS14 treated animals.

SFig.1: **A** distribution of all ripples across clusters (all events). **B** ripple fractions per cluster per post trial perioSfigd **C** count of events per type and post trial period, including TST (Total Sleep Time, in minutes) shown on the right axis. **D**. Counts and **E**. Rates of ripple types per animal (each including post trial periods), **F**. rates of ripple types per condition (including post trial periods). **G.** characteristics of ripple types based on new PCA run on the ripple filtered (100-250 Hz) and not raw traces.

**SFig. 2 Sequences and Couplings:** A. For each cluster type (including post trial periods as n) the different sequences and their occurrence rates split for conditions. All types of clusters increased DR coupling in the complex memory conditions and ripple during spindle (RwS), spindle with at least one ripple (SwR, e.g. multiple RwS could be one SwR), and the same with a preceding delta (DSwR) all increased for simple and complex learning. Findings already reported in [4]. B. Delta followed by ripple and ripple during spindle split for Clusters but only for the subselection of events used in the granger causality analysis (including post trial periods). C. Fraction of delta and spindle (left, right respectively) that were coupled to their hippocampal or cortical counterparts considering DR and RwS events. For DR C2 and C3 showed higher cortical to hippocampal coupling than medium-sized (simple D-D and S-S events, F_2, 501_=57.8 p<0.0001 interaction brain area cluster ANOVA, tukey posthocs, including post trial periods) and for spindle with ripples only C3 showed this increased coupling of cortical events (F_2, 503_=55.3 p<0.0001 interaction brain area cluster ANOVA, including post trial periods). D. Latencies of these coupled events (including events). The plots A, C, D and E incorporate all events from the vehicle dataset. E. Latencies of ripple type succession. C3short-C2 latencies had a more distinct peak at the expected doublet/multiplet interval. All error bars SEM

**SFig. 3 Spectrograms and Granger Causality Analysis** A and B for 100-300 Hz.

**SFig. 4** A. Cluster space including Cluster 4, B and C Ripple-PCA in Veh and RGS14 clusters 1-3 individually.

**SFig. 5** Ripple-PCA in RGS14 with and without clusters (A.). B. Rates for each cluster with pink shading for RGS and C. Granger values split for pre and post ripple.

**SFig. 6** Behavior during A, Overlapping (complex learning) and Simple Learning. For each Exploration time (i) and discrimintation index (ii) during the five training trials, as well as discrimination index at the 24h test trials for different time periods within the test trial (cummulative). Discrimination index is calculated (time spend exploring moved object – time spend exploring stationary object)/ total exploration time. C. 24h and 72h Test for Simple and Complex (Overlapping) of behavioral animals, difference between RGS14 and vehicle is visible in the 72h Test, where they have increased interference effects. See[4]. Animals with electrophysiological implants did not have the 72h retest.

**SFig. 7** Granger analysis as in Fig. 3 but with data set balanced across animals instead of closest to median.

**SFig. 8** Histological verification of RGS14 overexpression, from left to right DAPI, anti-RGS14 and overlay for top intervention group and bottom control same area.
